# Epidemic Erysipelas

**Published:** 1847-10

**Authors:** H. Jewett

**Affiliations:** Allen’s Hill, Ontario Co., N. Y.


### BUFFALO MEDICAL JOURNAL

AND

### MONTHLY REVIEW.

VOL. 3. OCTOBER, 1847. NO. 5

### ORIGINAL COMMUNICATIONS.

ART. I.—*Epidemic Erysipelas*. By H. Jewett, M. D., Allen's Hill, Ontario Co., N. Y. Being an address, read at the annual meeting of the Medical Society of Ontario County, July 1847.

[*Communicated for the Buffalo Med. Jour., by request of the Society.*]

The history of Epidemic diseases is a subject which has attracted the attention of medical writers from the earliest ages. If we glance at the records of the past, or recur to our own experience and observation, we perceive that at all times, and in all countries, many diseases have assumed an epidemic character, and prevailed with greater or less mortality. Fevers, dysenteric affections, cholera, and various other diseases, have taken this peculiarity, and spread over entire countries; then, again, assuming the endemic aspect, they have been confined to a limited region, prevailing with different degrees of malignity in certain seasons, situations, and in particular states of the atmosphere. We see them springing up in different parts of the country almost simultaneously, without any connection, without obvious producing causes, other than continued cold, heat, or moisture, or the influence of all these combined. And in no disease have we seen the truth of this remark more clearly exemplified than in the prevalence of the malignant or epidemic erysipelas in this country. We may speculate and theorize upon the remote and proximate causes of this malady : but our speculations and theories are all in vain. Theories may be profitable in the development of truth, but when they are made the basis of practical error, they become dangerous to human life. It is truth, experimental truth, that we require to guide us in the recondite and intricate paths pertaining to our organization, and in the adoption of means best adapted to the preservation of life, and the promotion of health.

Many diseases that are strictly epidemic, are by some considered contagious, from the fact that they prevail to so great an extent in hospitals, neighborhoods, and circumscribed sections. But all must be aware of the difficulty in distinguishing between diseases that are communicated from one person to another, and those which at the same time affect many individuals who happen to be placed in similar circumstances, and are subject to the same external influences. In speaking of the non-contagiousness of disease, I would be understood to use the term in a modified sense. Any cause that produces a depressed and debilitated condition of the patient's constitution—exposure to damp, fatigue, severe and continued cold, fear of sickness, in short, any thing which tends to disturb the natural equilibrium of the system, doubtless predisposes to disease, and that predisposition is materially increased, and the character of the disease established, by coming in contact with, and inhaling the efluvia arising from a person laboring under any prevailing epidemic, and more especially so, if the epidemic puts on a malignant character. A whole community, or neighborhood, during the prevalence of an epidemic, seems to be charged with some mysterious morbific agency, which is developed and brought into action by external influences. We have recently seen this in the prevalence of the typhoid fever in the town of Richmond, in this county. Commencing at the outlet of the Honeoye Lake, and extending along the bottom lands contiguous to the stream, it affected, all more or less within the sphere of its influence. The disease in this instance put on a peculiar and specific character. Most of those who were in contact with the sick, or in occasional attendance upon them, suffered, to some extent, from the combined influence of watching, fatigue, and direct exposure to the person laboring under the disease, superadded to atmospheric malaria.

It is a peculiar feature in epidemic diseases to render other affections less common during their prevalence. The predisposing causes, which must be some atmospheric condition, entirely inappreciable by our senses, or present means of investigation, seem to exert a specific influence to the exclusion of most other diseases.

We find, however, invariably puerperal peritonitis prevailing at the same time with epidemic erysipelas, which tends to prove some direct connexion, or dependence one upon the other, although in the puerperal cases there is no visible manifestation of the erysepelatous character, and no essential difference from ordinary peritoneal inflammation except in the rapidity and certainty with which the disease runs on to its fatal termination.

During the prevalence of the epidemic in Vermont and New Hampshire, in 1842–3, puerperal peritonitis prevailed with alarming fatality. In one county in Vermont 30 cases occurred, all of which (but one) proved fatal. In Bath, with a population of 1600, 20 women died of puerperal fever, and 40 of erysipelas. As these diseases exist at the same time, can their origin be traced to similar causes? or, if not, what connexion exists between them? Dr Allison remarks, that when erysipelas extensively prevails, puerperal fever becomes epidemic. Drs. Ramsbotham, Collins, Acherly, Abercrombie and Cusach bear testimony to the same fact.

In the history of the epidemic that visited Vermont, Massachusetts, and the eastern part of this State during the years of 1840–1–2–3–4, we have abundant proof to the same point. And I hazard nothing in saying, that every practitioner who has been engaged in treating the one, can bear painful record to the fearful and speedy mortality of the other.

These diseases are not in reality ordinary inflammation, they seem to possess a new, peculiar, and specific character. We see them occurring together epidemically, ceasing together without any obvious reasons, and suddenly and strangely reappearing. The truth of the statement has fallen under the personal observation of most of us; and for its explanation we are compelled to allow some powerful predisposing cause, upon which depends the development of both erysipelas and puerperal fever. This unknown cause is brought into action by a variety of external influences. Dr Gordon says, "they are concomitant epidemics." That there is a close connexion between them, an intimate dependance, the one upon the other, we have the most convincing evidence, in the history of their ravages in our own neighborhood, as well as in other parts of the state. In the immediate neighborhood when the fever was common, scarcely a case of lying-in escaped its deadly influences; and in all the cases of puerperal disease that have occurred, there has been great uniformity of time, character, and symptoms—unlike erysipelatous fever as it prevails in other forms, and under different circumstances, where we find every grade of morbid influence exists, from the simple sore throat, to the most malignant typhoid condition. It is a question full of interest to the Physician, and to community, whether there be any connection, dependence, similarity in origin, mode of propagation, or in any of the essential features of these diseases. The question forcibly suggests itself, does contagion or infection contribute to the production of puerperal fever? If not, how does it happen that the proportion of such cases is greatly increased during the prevalence of the epidemic? Then again, the facts are too numerous, and stubborn, to be resisted, that the physician, or nurse, may be, and there is too much reason to believe frequently is the medium of communicating the disease.

Caution, seclusion, or living out of the region of the affected atmosphere, are not safeguards, if the patient be attended during labor by an accoucheur engaged in treating subjects laboring under this epidemic—and here allow me to cite one case in corroboration of this fact:—

The patient was the wife of an intelligent medical friend in an adjoining town, who, suffering from an attack of pulmonary hemorrhage, requested one of his medical bretheren to attend upon her during her confinement. The physician, at the time, was extensively employed in treating erysipelatous affections, which prevailed in another town with alarming fatality, and at the moment of the summons was engaged in dressing an abscess, the result of phlegmonous erysipelas. The patient was delivered of her second child in the ordinary time, and left with a promise of a speedy convalescence. In 49 hours from the time of the delivery, she was seized with a severe chill, and died, at the end of the fourth day, of puerperal fever. Previously to this time, no cases of the disease had occurred in the neighborhood, or town; neither had any tendency of the kind manifested itself. But from that date erysipelatous affections became common.

Two important facts are to be deduced from the foregoing case: 1st. that the disease is communicated by the clothing, or the actual contact of the hand under such circumstances; and 2d, that the phlegmonous, or the erythematous form of the disease grows out of puerperal inflammation. Numerous other instances that have come under our notice, might be adduced, presenting the most unequivocal testimony that the disease may be communicated by contact to parturient females.

I might here remark, that at the time of its prevalence in the Western part of the County, and during my attendance upon the disease, the greatest precaution was taken, to remove, as far as possible, by repeated washing, and change of clothing, all danger of infection from my person, before venturing to the sick room of a patient in child bed. During its prevalence in the fall and winter of 1845, at Honeoye, in almost every instance of lying-in in that region, the patient fell a victim to the disease; and so deeply impressed were the citizens of the danger, that all parturient females were removed, by the judicious advice of the attending physicians, to the neighborhood of a medical man without its fatal influence; and I believe in every instance there was an exemption from the disease.

In a history of erysipelatous fever that prevailed in New Hampshire and Vermont in the spring of 1842, given by Hall and Dexter, in the January No. of the American Journal of the Medical Sciences, we have one of the first condensed accounts of its fatal ravages in an epidemic form. Here, as elsewhere, the fact is set forth that lying-in females almost indiscriminately were ready victims to the disease. As before stated, in Caledonia Co., Vermont, thirty cases of puerperal peritonitis occurred, only *one* of which recovered; and in Bath, N. H., twenty mothers died of the same disease, and forty of erysipelas.

Almost simultaneously we find accounts of its springing up here and there in different parts of the country. In the eastern and central portions of this State we heard of a few sporadic cases, but it attracted little attention until the fall of 1844, when it made its appearance in the Counties of Thompkins, Cayuga, Cortland, and Seneca, as an epidemic, an account of which will be found in the New-York Journal of Medicine for July, 1846. We also find statements of its ravages in Canada as early as the summer of '41. In the winter of 42–3, it prevailed in Missouri, with unusual mortality, during the cold season. In a history given of it by a correspondent, the more mild cases commenced with lassitude and depression, loss of appetite, slight chill succeeded by slow fever, thirst, red tongue, fullness in the head, &c. Then, again, the attack was violent, the chill continued until the patient sunk under the force of it.

In a large proportion of cases the throat was the principal seat of the disease, swelling to such an extent as to render deglutition almost impossible, and greatly embarrassing respiration, the face becoming livid and œdematous, the pulse feeble and tremulous, extremities cold, and in this condition the patient frequently sinks; but if he survives twenty-four or thirty-six hours, the swelling of the throat subsides, the breathing and swallowing become more free, and, unless the disease is transferred to some vital organ, the patient recovers. The disease in that climate runs on much more rapidly to its termination than here, where we have a lingering, protracted convalescence.

In November, 1843, the epidemic showed itself in the Western part of this State, in the town of Wales, Erie Co. For its history and treatment I am indebted to an intelligent medical friend residing and practicing there at that time, and who attended the first cases that were distinctly characterized as malignant erysipelas.

The attack was usually ushered in by a slight chill and soreness of the throat, tongue red, and covered with a viscid slime—the pulse small and rapid, ranging from 120 to 150 in a minute. In some instances death ensued from the extreme swelling and effusion about the throat, producing suffocation. In about one third of the instances it manifested itself by external inflammation upon the face more than elsewhere, but no part or structure was secure against its attack. The inflammation was generally confined to the skin and cellular membrane, and in many instances it extended to the subjacent and intermuscular structures, forming deep and extensive abscess. Here, again, we have to record the melancholy fact, that females who had recently become mothers were swept off almost indiscriminately. In the town of Wales there were 300 cases. and 82 deaths, 27 of which were lying-in females, and one only survived.

In the town of Holland there were 203 cases, and a smaller proportionate number of deaths. In the town of Bennington sixteen parturient females died; in Alden, Java, and Shelden, the disease spread, but in a less malignant form.

About this period we heard of its ravages in the Counties of Genesee, Wyoming, and Allegany. In the towns of Alexander, Castile, and Nunday, it was peculiarly fatal. In December, 1844, the first cases showed themselves in Lima, Livingston Co., where it prevailed for six months with great mortality. I am greatly indebted to my professional friend, Dr. Whitbeck, for the following statistics, as well as for the history and treatment of several cases which fell under his observation. In a communication from the Dr. on that subject, he says: "there were seventy cases of the worst grade of the disease, that came whithin my notice, besides a great number of milder ones that were not worthy of particular attention. Of this number, there were 25 deaths—15 puerperal cases, 12 deaths, in eight of which the disease located upon the thoracic viscera—abdominal, not puerperal, 7—head 27—extremities 16.

For several months previously to its showing itself in an epidemic form in the western part of this County, tonsilitis and apthous sore throat were exceeding common. Indeed, all the cases of disease that occurred, were modified by the peculiar anginose character, without external erysipelatous inflammation, or severe internal congestion. A few sporadic cases, putting on the erysipelatous character, occurred in the spring of 1845, which excited no apprehension among the citizens, as the nature of the malady was not distinctly understood. They were, however, the foreshadowing of the disease in its epidemic form, which produced consternation and alarm wherever it appeared.

The first and only well marked case, at that time, happened in April, 1845, in the person of Mrs. M., aged 32, of delicate nervous temperament. After a few days of slight indisposition, she was seized with intense pain in the head, small thready pulse, cold extremities, and stolid countenance, with intolerance of light. The intensity of the pain induced me to open a vein at once, which afforded immediate relief from the severity of the suffering. Cold applications were made to the head and sinapisms were applied to the feet, ankles and wrists. The next day reaction was fully established,—full strong pulse, burning heat of the skin, and dry red tongue. It was not, however, until the fourth day that the nature of the disease was fully developed by a deep scarlet blush upon one ear, spreading over the temple, accompanied with soreness of the throat, and extreme tenderness of the scalp upon the side inflamed, During the first four days it was looked upon as a case of ordinary meningeal inflammation. So soon as the nature of the case was clearly revealed, the mild alterative and antiphlogistic plan of treatment was pursued, together with surrounding the inflamed part by cauterizing with the nitrate of silver. To allay the burning heat of the skin the inflamed part was frequently moistened with a tepid solution of murias ammoniæ, which afforded the patient some relief, without repelling the eruption. The type of the disease in the onset was decidedly inflammatory, yet early indications of sinking forewarned me of danger from another source, and the necessity for support, and the sustaining regimen. The convalescence in this case was extremely slow, under the influence of mild tonics, doubtless owing to the active treatment necessarily pursued at the commencement, to avert the danger that threatened the brain. The details of this case have not been given, but only some of its prominent features, inasmuch as it was six months in advance of the epidemic that followed.

In November, 1845, the disease showed itself epidemically at Honeoye, Ontario Co., and for some time was confined to that locality. The first cases that appeared were attended with swelled throats, and active peritoneal inflammation, which in many instances terminated rapidly in gangrene and death. In other instances the face and scalp swelled enormously, attended with most threatening indications. It was soon accertained that if it was not complicated with visceral disease, the danger was comparatively slight, and extended inflammation was looked upon as the most desirable form that the disease could present. For a time it was confined to the valley of the Honeoye lake; but it spread to the adjoining highlands, where it prevailed during the latter part of Winter and Spring. An isolated case would suddenly spring up in a hitherto uninfected district, and almost immediately the whole neighborhood would be seized, some with only slight soreness of the throat, others with violent organic inflammation, which latter cases were uniformly complicated with the anginose condition of the fauces, and in about one third of the instances the disease would be characterized by the peculiar erysipelatous swelling upon the face or extremities. The most alarming form that the disease could present, was that of peritoneal inflammation, most sudden and violent in its inception, rapid in its progress, and fatal in its consequences. Another condition peculiarly alarming, was when the mucous membrane of the fauces was highly inflamed; instead of speading along the nasal passages upon the exterior of the face, it extended by continuity of structure to the lungs, attended with oppressed and labored breathing, expectoration of a dark, bloody, tenacious mucous, great prostration of the vital powers, rapid pulse, and dry red tongue. In such instances, if the patient had passed the meridian of life, or possessed a feeble constitution, he almost inevitably sunk, before means could be used to arrest the rapid and fatal progress of the disease.

In all there were about 150 well marked instances in the vicinity of Honeoye, and twenty deaths. The proportionate number of deaths was much greater at the commencement of the epidemic than after it had prevailed for a time, and it is worthy of remark, (and the fact is borne out by observation wherever the disease has appeared) that the first few cases were more malignant and fatal than subsequent ones. The disease generally disappeared by showing itself in diminished violence, until the only evidence of its existence was a slight soreness of the throat, with those who had not been previously affected. I have transcribed a few cases from some hasty notes made at the time, but unfortunately in not a single instance of mortality was post mortem examination made, during the prevalence of the disease in the western part of this county. In some of the more malignant instances decomposition commenced in some parts before the circulation entirely ceased, and at the end of six hours great difficulty was experienced in placing the body in the coffin, so rapidly did the putrifying process take place, rendering speedy interment indispensably necessary. This, together with the fatal consequences to some who have ventured the investigation, deterred from prosecuting pathological inquiries upon the dead subject.

Mr. W., aged 44, of pulmonary diathesis, was seized on the 4th of April 1845, with violent chill, pain in the head and back, and was generally in great distress. I saw him soon after the reaction came on, the pulse 120, full and resisting, face flushed, restlessness accompanied with pain in the side, and great difficulty of swallowing, which came on suddenly during the first chill.

The case presented nothing unnsual from the prevailing sore throat, except the suddenness and intensity of the attack. The erysipelas had not made its appearance in this immediate neighborhood, yet most of the diseases throughout the region were complicated with inflammation of the fauces.

Took 12 oz of blood from the arm with immediate relief to the head—gave proto. chlor. hydrarg. grs. 20, to be followed with ol ricini if necessary. Saw him on the 5th, no material change except a slight abatement of the symptoms. Applied a blister to the throat, and gave sudorifics and anodynes, which were continued on the 7th.

On the morning of the 8th, one ear was discovered tobe swollen, red, and extremely tender to the touch—pulse 120, skin dry, tongue red in the centre with yellowish buff upon the sides, mucous membrane of the fauces highly inflammed.

Tenth, redness and swelling extending upon the temple and forehead, pulse rapid, tongue dry and rough, with depressed centre and elevated edges. Seared the skin with strong tinct. iodine in advance of the inflammation, to prevent its spreading over the face, and penciled the swollen part with a more dilute tinct. of the same. Gave proto. chlo. hydrarg. grs. 2, ipecac. gr. 1, sulph. morph. 1-6 gr. every four hours. 12th, inflammation had not overstepped the mark set by the iodine, but had spread in other directions over the scalp—pulse 125, compressible, somewhat irregular, mind wandering. ℞. spt. mindereri oz. ½, quinine grs. 2, alternated every four hours.

Thirteenth, ear enormously swollen and vesicated, inflammation had reached over the entire scalp; subsultus, coma, incoherency increased, pulse small, 130 per minute. ℞. quinine, grs. 4, camphor 2 grs. carb. ammon. 6 gr. combined, and alternated every four hours, with infusion of valerian and serp. virg. scalp kept wet with tepid solution of sulph. ferri.

Fourteenth, appearance much the same, treatment continued.

Fifteenth, passed a more quiet night, pulse 110, more firm and steady, consciousness improved, swelling of the ear first affected diminishing, the other ear and side of the head much swollen, with a collection of pus over the mastoid process.

Sixteenth, general appearance somewhat improved, tongue less dry, mind more clear. when fully aroused, redness of the side of the head first affected diminishing, pulse 106 in a minute. ℞ quin. grs. 3, carb. ammon, 6 gr, every six hours, alternated with infusion serp. virg. and rad. valerian.

Eighteenth, condition much improved. Gave infusion of cortex peru., serp virg. occasionally. From this time the convalescence gradually went on, and the patient recovered.

Case 2d; Mr. A., aged 60. Of natural strong constitution, but intemperate habits. Saw him for the first time on the 13th of April, 1846; had returned the day previous from a usual debauch of a week's continuance—was taken during the night with severe chill. Saw him in the morning after reaction came on—complained of difficulty of swallowing, mucous membrane and glands of the throat highly inflammed, pulse 100 with fulness of the artery, but easily compressed, tongue red and somewhat dry. Applied fomentations to the neck, and gave proto. chlor. hydrarg, grs. 4, ipecac ½ gr. every four hours, until the bowels were evacuated.

Fourteenth, countenance hippocratic, pulse intermitting, bronchial tubes filled with a bloody, tenacious mucus, which was expelled with great difficulty, somewhat comatose, slow labored respiration. ℞. quinine grs. 4, carb. ammon 6 grs., camph gr. 1, every four hours, altrenated with influsion of serp. virg.

Fifteenth, symptoms greatly aggravated, and the patient died on the evening of the third day, evidently from the inflammation and consequent effusion about the throat and upper portion of the lungs.

Case 2d; Mrs A., aged 23. Of good constitution, was taken on the 1st of June, 1846, with general lassitude, pain in the head and back, chilliness followed by feverish reaction. Saw her on the 11th, face flushed, skin hot and dry, pulse 120 small and hard, tongue red and covered with a slimy mucus—gave her Hydrarg, C. Creta gr. 10, Ipecac, gr. 1, every four hours, until the bowels were evacuated ; directed sinapisms to the extremeties. 12th, medicine had acted desirably upon the bowels, pain in the head intense, flushed face, full hard pulse. Venesection, 12 oz. with immediate relief to the head, applied epispastic to the neck, gave Proto Chlor. Hydr·, 2 gr., Ipecac, 1 gr., every 4 hours.

13th, Bowels moved twice during the twenty-four hours, pain in the head abated, pulse, 100, compressible, general symptoms all improved. 14th and 15th, convalescence continued—able to sit up and walk about the room ; medicine discontinued. At 9 o'clock on the morning of the 16th, complained of pain in the umbilical and hypogastric region. Saw her at 10 A. M.—slept well during the previous night, pulse somewhat accelerated, small, but not hard, tongue moist, and comparatively normal in appearance—had in the morning, a free, natural evacuation of the bowels, which were not tumid, but slightly tender on firm pressure. Saw nothing in the condition of the patient indicating danger, was able to walk about her room. Gave Pulv doveri, 2 gr., morphine Sulph. 1–6 gr.. to be repeated every three hours if the pain continued. Applied mustard paste to the bowels. Was summoned at 9 in the evening to see the patient. The messenger remarked, "there was something singular in her appearance," and, to my astonishment on arriving, found her in *articulo mortis*—cadaverous countenance, surface bathed in a cold, clammy sweat, cold, pulseless extremeties, occasional vomiting, and continued hiccough.

On inquiry, the pain in the bowels had increased, and been intense through the day. Stimulants external, and internal, exerted no influence, and she died at 4 o'clock on the morning of the 17th, with all the indications of gangrene of the peritoneum, twenty hours from the relapse, and the 6th day from the attack. On critical inquiry of the nurse she remarked, that from the time the pain left the head, she had complained of an unnatural, uneasy sensation in the bowels. But my attention had not been directed to it by the pulse or condition of the tongue, and there was every indication of convalescence up to the morning of the 17th.

The father and mother-in-law were both sick in the house at the time with malignant Erysipelas, the one with extensive phlegmonous abscess of the calf of the leg, the other with Erysipelatous inflammation of the throat, extending to the face and scalp, closing entirely the eyes, and obstructing the respiration and deglutition, presenting one of the most loathsome and malignant instances I ever witnessed, death ensuing on the 9th day.

There are some features in this disease, worthy of being noted ; the individuals attacked being adults—children generally escaping —and the rapid transition of inflammation from one part of the body to another, leaving the original point of attack nearly free from disease, and the part secondarily seized being most severely affected and often fatally disorganized. The most fearful and dangerous seat of the disease is the fibro-serous membranes in the interior of the body ; and the prognosis is decidedly unfavorable, when the peritonum, pleura or membranes of the brain are involved.

Numerous other instances might be adduced to show the varied and changing nature of this epidemic, but the limits of this paper preclude any farther trespass upon your time, and I will barely allude to the general principles of treatment adopted in different localities where the disease has prevailed.

The remedies instituted have been as various and diversified as the opinions concerning its nature and cause, and consequently, every plan and nostrum that ingenuity could invent, have been brought into requisition. General and indiscriminate bleeding, was by many adopted, and was, as might have been predicted, followed by general and indiscriminate slaughter. Calomel in large doses, and small doses has been resorted to, and yet the disease has marched on to its fatal issue. The high pressure Thompsonian hot drop system has been applied, and yet they died! Mammoth doses of Quinine have been administered, and the result has not been materially changed. Homœopathy, with its sugar pellets, and infinitessimal doses, has been invoked, and through the mysterious agency of this infinitely wonderful system, some valuable hints may be deduced.

A great majority of cases require only mild treatment. Active evacuents of all kinds are to be used with great caution. Early bleeding, only, if at all, should be practised, and that when the disease is complicated with Meningeal Inflammation, or a strong determination to the lungs, with full and hard pulse. In any case, great discrimination and care in the use of the lancet, must be exercised.

In the puerperal cases just reported, in the town of Wales, Erie Co., 25 of the 27 were bled, *all of whom died*, On the contrary, the too early use of tonics and stimulants are to be deprecated. They are to be kept in reserve, and promptly introduced on the first indication of waning of the vital energies. The more violent cases of Erysipelas, like typhus fever, have an inflammatory stage, followed by collapse, and our efforts should be directed to avert the danger arising from this last condition. The great object of equalzing the circulation, and restoring the vital energies, has been effected by sudorific remedies alone.

Warm, mild teas drank freely, in connection with heated bricks wrapped in wet cloths, and applied externally, have had the effect to bring on profuse perspiration, and thereby relieve the local congestion, and diminish the duration and violence of the disease. This treatment, followed by the use of alteratives, diaphoretics and anodynes, has been more successful than the use of more active remedies.

Blisters, tincture of Iodine, Nitrate of silver, solution of the sulphate of iron, and muriate of ammonia, are among the common external local applications that have been recommended, and used with various opinions as to their efficiency and value.

Notwithstanding the many theories and facts that have been elicited by the prevalence of this disease, we are forced by sad experience to acknowledge, that many cases of this formidable Epidemic have thus far bid defiance to all remedial agents in our possession, and we are compelled to witness the impotency of our art in staying the progress of death.

